# An overview of anti-drowning systems in cars

**Published:** 2024-10

**Authors:** Azar Darvishpour, Shiva Mahdavi Fashtami

**Affiliations:** ^*a*^ Social Determinants of Health Research Center, Trauma Institute, Guilan University of Medical Sciences, Rasht, Iran.; ^*b*^ Zeyinab (P.B.U.H) School of Nursing and Midwifery, Guilan University of Medical Sciences, Rasht, Iran.; ^*c*^ Master of Internal-Surgical Nursing, Pirouz Hospital, Guilan University of Medical Sciences, Rasht, Iran.

## Abstract

**Background:**

Overturning vehicles is a significant threat that leads to the unexpected death and loss of its occupants. Most of these deaths are preventable. For this reason, it seems necessary to focus research on the development of anti-drowning systems to prevent deaths in water-related accidents. The current research was conducted to identify anti-drowning systems in cars.

**Methods:**

The present study is a systematic review. In order to collect data, English articles available on Web of Science, Google Scholar, PubMed, and Scopus databases were reviewed from January 2000 to July 2024 with the keywords “Review”, “Drowning”, “anti-drowning systems in cars”, and “cars' drowning”.

**Results:**

Among 82 articles, after excluding unrelated studies, five studies that met the inclusion criteria were analyzed. Several systems were identified from the analysis of these studies. 1- Automatic window opening system for vehicles immersed in water when the vehicle is vertical and facilitates quick escape for the passenger. 2- Drowning prevention device using the BeiDou navigation satellite system to monitor vital signs and send drowning location information 3- Puddle Jumper system that provides a vehicle water level monitoring and control system to alert drivers from the current water level out, and activates a mechanism to raise the vehicle if the water level rises dangerously. 4- Lane departure warning systems (LDW) or lane keeping systems (LKS), which warn or steer back to vehicles that are likely to leave their lanes. 5- Installing safety devices such as external airbags as an option to increase the vehicle's buoyancy and prevent it from sinking.

**Conclusions:**

Considering the limited number of studies in the field of anti-drowning systems in cars and the necessity of conducting more research, encouraging cooperation between researchers, car manufacturers, and industry experts is vital for the continuous development and implementation of anti-drowning systems in cars.

**Keywords:**

Drowning, Anti-drowning systems, Cars, Review

